# Supplementary material for: Zeaxanthin Mitigates Hydroxychloroquine‐Induced Retinal Toxicity and Metabolic Reprogramming in ARPE‐19 Cells
Source: J Biochem Mol Toxicol. 2026 Jul 8;40(7):e71015. doi: 10.1002/jbt.71015 (PMC13347105; doi:10.1002/jbt.71015)
Supplement: Supplementary file 1 — Figure S1: Dose‐dependent effect of HCQ on ARPE‐19 cell viability after 72 hours of treatment. Cells were exposed to various concentrations of HCQ (15.6–1000 µM), and viability was assessed by MTT assay. Data are presented as mean ± SE (n = 3). [file JBT-40-e71015-s001.docx]

**Zeaxanthin Mitigates Hydroxychloroquine-Induced Retinal Toxicity and Metabolic Reprogramming in ARPE-19 Cells**

**Running title:** Zeaxanthin Prevents Hydroxychloroquine-Induced Retinal Toxicity

**Münire Berna Asal Altıparmak^1^, Emine Merve Yıldırım^1^, Ozan Kaplan^2^, Emine Koç^2,3^, Açelya Erikçi^4^, Mustafa Çelebier^2^, Tuba Tüylü Küçükkılınç^1*^, Gülberk Uçar^1^**

**^1^** Department of Biochemistry, Faculty of Pharmacy, University of Hacettepe, Ankara, Turkiye

**^2^** Department of Analytical Chemistry, Faculty of Pharmacy, University of Hacettepe, Ankara, Turkiye

**^3^** Department of Medical Biochemistry, Graduate Institute of Health Sciences, İnönü University, Malatya, Türkiye,

**^4^** Department of Biochemistry, Faculty of Pharmacy, University of Lokman Hekim, Ankara, Turkiye

**^*^Corresponding Author:** Tuba Tüylü Küçükkılınç, Department of Biochemistry, Faculty of Pharmacy, University of Hacettepe, Ankara, Turkiye

**Email:** [ttuylu@hacettepe.edu.tr](mailto:ttuylu@hacettepe.edu.tr)

**ORCID:** 0000-0003-1566-0717

**Acknowledgement:** This study was funded by the Hacettepe University Scientific Research Coordination Unit Project ID: TUK-2023-20817. This study is related to the Ph.D. thesis of Münire Berna Asal Altıparmak.

**Abstract**

**Background:** Long-term usage of the commonly used autoimmune disease drug hydroxychloroquine (HCQ) can result in retinal toxicity due to elevated lysosomal pH, mitochondrial damage, and oxidative stress. Zeaxanthin (ZEA), a carotenoid found mostly in the macula, is known for its antioxidant and cellular protective properties. The purpose of this study was to examine how ZEA would lessen the cytotoxicity, oxidative stress, mitochondrial dysfunction, and metabolic reprogramming driven by HCQ.

**Method:** ARPE-19 cells were treated with HCQ (1 mM), ZEA (1–10 μM), or their combinations for 72 hours. Cell viability (MTT), apoptosis/necrosis (Annexin V–7AAD), reactive oxygen species (DCFDA), mitochondrial membrane potential (TMRM), and lysosomal acidification (Acridine Orange staining) were analyzed. Additionally, LC–QTOF/MS-based untargeted metabolomics analysis and pathway enrichment assessments were performed.

**Results:** HCQ treatment decreased cell viability to 23%, increased necrosis and ROS production, and led to mitochondrial hyperpolarization and lysosomal neutralization. Metabolomics analysis revealed a decrease in taurine, GABA, uric acid, and TCA intermediates, and an increase in palmitic acid and prostaglandin levels. Combination treatment with ZEA (10 μM) increased cell viability to 47%, reduced ROS and necrosis rates, and increased pantothenic acid, γ-glutamyl cysteine, and citrate levels, supporting glutathione synthesis and phase II detoxification pathways.

**Conclusion:** Zeaxanthin demonstrated a significant protective effect on HCQ-induced oxidative, mitochondrial, and metabolic stress. These findings suggest that ZEA may be considered as a potential therapeutic agent against HCQ-induced retinotoxicity.

**Keywords:** Hydroxychloroquine, Zeaxanthin, ARPE-19, Metabolomics, Mitochondrial Dysfunction

**1 INTRODUCTION**

Hydroxychloroquine (HCQ) is a widely used drug for the treatment of autoimmune diseases and malaria; however, long-term use can lead to serious side effects such as retinotoxicity (1). HCQ has been reported to block autophagy by increasing lysosomal pH, resulting in mitochondrial damage, reactive oxygen species (ROS) accumulation, and cell death (2). Retinal pigment epithelial (RPE) cells are a critical layer maintaining photoreceptor homeostasis, and the ARPE-19 cell model is frequently used to study these toxicity mechanisms in vitro (3).

Autophagy inhibition leads to mitophagy failure, leading to metabolic reprogramming such as changes in mitochondrial membrane potential (ΔΨm) and suppression of the TCA cycle (4, 5). In the literature, HCQ has been shown to cause a decrease in metabolites such as taurine and γ-aminobutyric acid (GABA), a loss of uric acid in purine catabolism, and interference with lipid metabolism(6-8). These changes may accelerate retinal degeneration by triggering oxidative stress and necrotic cell death.

Zeaxanthin (ZEA), along with lutein, is a major component of macular pigment and has antioxidant and anti-inflammatory properties (9). ZEA is known to increase glutathione biosynthesis and induce phase II detoxification enzymes by activating the Nrf2 pathway(10); however, its effects on HCQ-induced retinotoxicity have not been investigated.

In this context, we evaluated the cytotoxic effects of HCQ in ARPE-19 cells using biological (cell viability, apoptosis/necrosis, ROS, ΔΨm, lysosomal acidification) and metabolomic analyses to determine the protective potential of ZEA against these effects. This study aims to provide new insights into the use of ZEA as a potential compound for the prevention of HCQ-induced retinal toxicity.

**2 MATERIALS AND METHODS**

**2.1 Cell Culture and Cytotoxicity Assay**

Hydroxychloroquine sulphate (Cayman,17911), Zeaxanthin (Cayman, 10009992), and DMSO (Emplura, Merck) were obtained commercially. ARPE-19 cells were commercially purchased (CRL-2302, ATCC, Manassas, VA, USA). The cell line was cultured in Dulbecco's Modified Eagle Medium/F12 (DMEM/F12, ATCC 30-2006) supplemented with 100 µm/ml penicillin (Sigma-Aldrich, St. Louis, MO, USA) and 10% Fetal Bovine Serum (FBS; Gibco/BRL, Gaithersburg, MD, USA) at 37°C and 5% CO_2_. Cells were checked every 2-3 days with an inverted microscope and passaged by dilution when they reached 75-80% density.

Cells were seeded in 96-well plates at 5 x 10^3^ cells/well and incubated for 24 hours to ensure adherence. To determine the appropriate HCQ concentration, preliminary dose-response experiments were performed with HCQ concentrations ranging from 15.6 µM to 1000 µM for 72 h. Based on these results, 1 mM HCQ was selected for subsequent experiments. The cells were then incubated with 1 mM hydroxychloroquine, 1-10 µM zeaxanthin, or combinations of these for 72 hours. In combination studies, HCQ was added 3 hours after the addition of ZEA, and MTT analysis was performed after 72 hours of incubation.

MTT assay was performed to assess cytotoxicity. Briefly, 10 µl of MTT reagent (5 mg/ml, Cayman 21795) was added to each well after incubation. The formed MTT formazan crystals were dissolved by adding 100 µl of DMSO, and cell viability was determined by comparing absorbance values at 690 nm (for reference wavelength) and 570 nm (for MTT formazan absorbance) using a BMG Omega microplate reader. IC50 values and viability percentages were calculated using GraphPad Prism 8.0.

**2.2 DPPH Assay**

The free radical scavenger 1,1-Diphenyl-2-picrylhydrazyl (DPPH) is used to track a chemical reaction that generates free radicals. Ascorbic acid was used as a reference antioxidant. Briefly, 50 μL of DPPH (200 μM) and 50 μL of the compound solutions at 200 μM were combined in a 96-well plate and incubated for 60 minutes at 37 °C in the dark. Using a multimode plate reader, the absorbance values at 517 nm were obtained (BMG Labtech Omega FLUOstar). The reduction percentage of DPPH was calculated by the following formula:

$$\%DPPH=1-\frac{Asample-Ablank}{Acontrol-Ablank}\times100$$

**2.3 Flow Cytometry Analyses: Determination of Apoptosis/Necrosis, Reactive Oxygen Species, and Mitochondrial Membrane Potential**

Flow cytometry enables the analysis of many parameters related to cell health, such as apoptosis, necrosis, cell cycle, and oxidative stress.

To elucidate cell death mechanisms, Annexin V-FITC/7-AAD Apoptosis Detection Kit (Elabscience, E-CK-A212) was employed following the manufacturer’s protocol. Briefly, 1 × 10⁶ compound-treated cells were centrifuged at 1200 × g for 5 minutes, washed twice with ice-cold PBS, and resuspended in 500 μl binding buffer. Cells were stained with 5 μl FITC-conjugated annexin V (10 mg/ml) and 5 μl 7-AAD (50 mg/ml) and incubated for 15 minutes at room temperature in the dark. Flow cytometric analysis was performed immediately. Cells positive for annexin V alone were classified as early apoptotic, while double-positive cells were identified as late apoptotic or necrotic.

ROS levels were assessed using the ROS Fluorometric Assay Kit (Elabscience, E-BC-K138-F) according to the manufacturer’s protocol. Briefly, 1 × 10⁶ compound-treated cells were centrifuged at 1200 × g for 5 minutes, washed with phosphate-buffered saline (PBS), and incubated with 25 μM DCFDA for 30 minutes at 37 °C in a 5% CO₂ incubator. Following incubation, cells were washed, resuspended in serum-free medium, and analyzed for fluorescence intensity by flow cytometry. Cells were also imaged under the ZOE Fluorescent Cell Imaging System.

Mitochondrial membrane potential was evaluated using the MitoProbe™ TMRM Assay Kit for Flow Cytometry (Thermo Fisher Scientific, M20036) following the manufacturer’s protocol. Briefly, 1 × 10⁶ compound-treated cells were centrifuged at 1200 × g for 5 minutes, washed with PBS, and resuspended in warm PBS or culture medium. Cells were then stained with 100 nM TMRM (diluted from the 20 µM stock in DMSO) and, where applicable, 50 µM carbonyl cyanide 3-chlorophenylhydrazone (CCCP) as a positive control for depolarization (added 5 minutes before TMRM). The mixture was incubated for 30 minutes at 37 °C in a 5% CO₂ incubator. Following incubation, cells were washed once with PBS and resuspended in 500 µl PBS. Flow cytometric analysis was performed, measuring fluorescence in the FL-2 or PE channel. Data were acquired from at least 10,000 events per sample, with mean fluorescence intensity (MFI) used to quantify ΔΨm changes.

**2.4 Acridine Orange Staining**

Acridine orange (AO) is a weak base (pKa 9.65) that accumulates in acidic lysosomes through protonation, limiting diffusion. Its fluorescence changes from green (monomeric, cytosolic) to red (dimeric, lysosomal), making it possible to measure lysosomal function and autophagy using fluorescence microscopy or flow cytometry(11, 12). AO staining was performed according to SenthilKumar(12). Briefly, ARPE-19 cells were treated with the indicated concentration of each compound or DMSO as a vehicle control for 72 hours. The cells were then washed with 1X PBS. 50 μl of 1 μg/ml AO was added to each well, and the plates were incubated at room temperature for 30 minutes. The cells were then washed twice (100 μl per well) with 1X PBS. 50 μL of 1X PBS was added per well, and the plates were then read on a BMG Omega microplate reader and visualized with ZOE Fluorescent Cell Imaging System. The level of lysosomal acidification was determined by normalizing the red signal (460/650 nm) to the green signal (excitation/emission: 500/526 nm).

**2.5 Statistical Analyses**

GraphPad Prism 8.0 software was used to evaluate data from in vitro studies (at least three replicates) for statistical significance. The mean ± standard error (SE) was used to express the results. One-way ANOVA was performed to evaluate differences. Dunnett's or Tukey's Multiple Comparison Test was used to compare groups against the control, and Bonferroni's Multiple Comparison Test was used for pairwise group comparisons. The threshold for statistical significance was p<0.05.

**2.6 Metabolomics Analyses**

**2.6.1 Metabolite Extraction**

Metabolite extraction, liquid chromatography quadrupole time-of-flight/mass spectrometry (LC/MS) analysis, and data processing were performed according to Kaplan et al(13). Control and treatment group cells were seeded in 60 mm plates as six biological replicates. Cultured cells were washed twice with phosphate buffer saline, and the cell lysis procedure was performed with liquid nitrogen. Then, 1 ml of cold methanol was added to each plate, and cells were scraped using a cell scraper. The collected lysate was transferred to a microcentrifuge tube. This process was repeated with 0.75 ml of methanol, and lysates were combined in the same microcentrifuge tube. Samples were centrifuged at 10.000 rpm at +4 °C for 20 min (Hettich Universal 320 R, Germany), and 0.8 ml of the supernatant was transferred to a new tube, then the solvent was evaporated using a vacuum centrifuge (Labconco, CentiVap, 7310030, USA). Samples were redissolved in 0.2 ml of acetonitrile: water (1:1, v/v). After centrifugation at 10.000 rpm at +4 °C for 10 min, 0.1 ml of the upper phase was taken into a glass vial and analyzed with a LC/MS device (Agilent Technologies, Q-TOF 6530, USA).

**2.6.2 Q-TOF LC/MS Analyses**

Injections were carried out via gradient elution with a reverse-phase chromatography column (2.1 × 100 mm, 2.5 µm, Xbridge, Waters, USA). The column temperature was maintained at 35 °C, while the autosampler was kept at +4 °C. The flow rate was set to 0.4 ml/min. The mobile phases consisted of water (phase A) and acetonitrile (phase B), both containing 0.1% formic acid. Analyte separation followed a gradient elution program: phase A started at 95%, decreased to 65% by the 2nd minute, 5% by the 8th minute, and returned to 95% by the 10th minute, followed by a 5-minute post-run phase. The injection volume was 10 μL. The mass spectrometry operated in negative ion mode with a scanning range of 75–1200 mass/charge ratio (m/z). Injections were performed in a randomized order, with extraction blanks and quality control samples injected every sixth run to ensure analytical consistency.

**2.6.3 Data Analysis**

After converting the raw data obtained from the Q-TOF LC/MS analysis into the mzML file format, data processing was performed using the MZmine software (20). In the MZmine program, filters such as m/z determination, chromatogram deconvolution, metabolite identification using the in-house library, filtering, and isotope grouping were applied to extract the peak areas of the data. During the evaluation phase, the relative standard deviation values of the quality control and pooled samples were first calculated. The matched metabolites were then filtered using extraction blank samples. To minimize errors arising from the analysis, each peak area was normalized by dividing it by the average of all peaks in the sample. Principal component analysis (PCA) and volcano plots were generated using all normalized peaks. Subsequently, t-tests and fold change values were calculated to compare groups based on the normalized values. Metabolite identification was performed using an in-house library (level 1 identification) containing 968 metabolites and putative matching (level 2 identification). The in-house library and putative matched metabolites were combined to generate a heatmap and variable importance in protection (VIP). Pathway analysis was conducted using metabolites with p<0.05 and fold change>1.5 from the putative and in-house library identified metabolites. This allowed the identification of altered metabolites and pathways between the HCQ, ZEA, combination, and control groups. The MetaboAnalyst (MetaboAnalyst ver 6.0) software (21) was used for PCA plots, volcano plots, heatmaps, VIP scores, and metabolite identification. The statistical significance threshold was set at p<0.05 and fold change>1.5. A Pearson correlation matrix was generated using the apoptosis (Annexin V/7-AAD), AO, DCFDA, and TMRM parameters after the metabolite fold change values were normalized using log2(|FC|) * sign(FC).

**3 RESULTS**

**3.1 Effect Of ZEA On HCQ-Induced Cytotoxicity In ARPE-19 Cells**

Preliminary dose-response experiments were conducted by treating ARPE-19 cells with increasing concentrations of HCQ (15.6–1000 µM) for 72 hours to determine the appropriate working concentration of HCQ. As shown in Supporting Figure S1, cell viability decreased in a dose-dependent manner. Treatment with 1 mM HCQ resulted in a substantial reduction in cell viability, providing a clear experimental window to evaluate the protective effects of zeaxanthin without causing complete cell death. Subsequently, the viability of ARPE-19 cells was evaluated using the MTT test following a 72-hour exposure to HCQ, ZEA, or their combination, as seen in Figure 1. Compared to controls, cell viability was drastically reduced to 23.2± 3.0% (p<0.0001) when 1 mM HCQ was applied alone. On the other hand, ZEA by itself had negligible effects on cell viability at concentrations between 1 and 10 μM; results ranged from 95.3 ± 5 % (10 μM ZEA) to 109.4 ± 8.0 % (1 μM ZEA). It was observed that at low concentrations (1–4 µM), ZEA did not significantly reverse HCQ-induced cytotoxicity, and the protective effect became more pronounced at higher doses, especially at 10 µM. In particular, cell viability almost doubled by 10 μM ZEA co-treatment in comparison to the HCQ group (47.4 ± 3.3%, p < 0.001), indicating ZEA’s protective effects. This finding suggests that the cytoprotective effect of ZEA may be dose-dependent.

Please insert Figure 1

**3.2 Effect of ZEA on Cell Death Mechanisms of HCQ Toxicity**

The ANX-AAD7 flow cytometry analysis was used to determine the viable, early apoptotic, late apoptotic, and necrotic cell populations in ARPE-19 cells after 72 hours of HCQ, ZEA, or combination treatment, as seen in Figure 2. The control group exhibited a healthy cell population, with a high viability rate of 95.46 ± 4.51%. There was minimal late apoptosis (3.49 ± 3.51%), necrosis (0.62± 0.61%), and early apoptosis (0.43 ± 0.40%). Administration of 1 mM HCQ significantly decreased viability to 53.40 ± 3.23% and increased necrosis to 43.18 ± 3.06% (p<0.0001). Late apoptosis decreased to 2.59 ± 0.18% and early apoptosis slightly increased to 0.83 ± 0.06%. This finding suggests that HCQ treatment causes a largely necrotic response.

In contrast, ZEA (10 μM) maintained a viability rate of 93.14 ± 1.21%, which was quite comparable to the control. Late apoptosis was 5.19 ± 1.07%, necrosis was 0.91 ± 0.21%, and early apoptosis was 0.77 ± 0.05%. These values demonstrate that ZEA has minimal cytotoxicity and maintains cell integrity. Treatment with 1 mM HCQ with 10 μM ZEA raised viability to 68.10 ± 2.52% compared to HCQ alone (p<0.0001). Necrosis decreased to 27.12 ± 1.28% (p<0.0001), late apoptosis increased to 2.75 ± 0.74%, and early apoptosis increased to 2.03 ± 0.83%. This revealed that ZEA reduced HCQ-induced necrosis while increasing cell survival, with no significant differences in early and late apoptosis rates.

Please insert Figure 2

**3.3 Effect of ZEA on HCQ-Induced Oxidative Stress**

In preliminary experiments, the antioxidant potential of ZEA was compared with that of ascorbic acid, a known hydrophilic antioxidant, using the DPPH radical scavenging test. At the concentration of 200 µM, ZEA scavenged 44.1 ± 3.1 % of DPPH radicals, while ascorbic acid showed a scavenging activity of 93.8 ± 1.6 % at the same concentration. These results demonstrate that ZEA exhibits a lower direct radical scavenging capacity compared to ascorbic acid in the DPPH radical scavenging test. However, considering the lipophilic nature of ZEA, it was hypothesized that it may possess complementary antioxidant potential in ARPE-19 cells, particularly through different mechanisms such as membrane protection and singlet oxygen quenching. Therefore, intracellular ROS levels were evaluated using DCFH-DA flow cytometry.

Utilizing DCFDA flow cytometry, the percentage of ROS-positive cell populations and geometric mean fluorescence intensity (MFI) were determined in ARPE-19 cells following 72 hours of treatment with HCQ, ZEA, and their combination (Figure 3). The control group exhibited a ROS-positive cell population of 12.1 ± 4.1%, demonstrating a stable oxidative status under normal conditions. The positive control (THBP) demonstrated higher MFI and a ROS-positive population of 34.2 ± 5.8%, indicating its significance as an oxidative stress inducer.

When compared to the control, ROS levels increased significantly after 1 mM HCQ was administered, revealing a ROS-positive population of 37.3 ± 1.7% (p < 0.0001). This aligns with the autophagy inhibition reported in the literature by HCQ, which may raise the production of ROS through mechanisms such as mitochondrial dysfunction and lysosomal stress(14).

Although ZEA (10 μM) alone slightly altered MFI, it also showed a ROS-positive population of 33.1 ± 1.9%. ZEA is known to have antioxidant or pro-oxidant characteristics, with varying effects depending on dosage and cell type(15, 16).

Cotreatment with 1 mM HCQ and 10 μM ZEA slightly decreased MFI values compared to HCQ, but increased ROS-positive population to 43.5 ± 4.1% (p < 0.001). The findings imply that ZEA either modifies the cellular distribution of ROS production through a different mechanism (i.e., lipid peroxidation) or somehow affects the intensity of ROS generation by HCQ. The fluorescent microscope images displayed in Figure 4 further support these conclusions.

Please insert Figure 3 and Figure 4

**3.4 Effect of ZEA on HCQ-Induced Mitochondrial Stress**

The alterations in ΔΨm of ARPE-19 cells were assessed using the TMRM flow cytometry technique following a 72-hour exposure to HCQ, ZEA, and their combination (Figure 5). MFI and the proportion of cells in the TMRM-positive population were calculated. The control group had a TMRM-positive cell population of 90.1 ± 2.1%, reflecting a balanced mitochondrial state under normal conditions. As expected, the TMRM-positive population was considerably decreased to 43.8 ± 1.8% by the positive control CCCP, a mitochondrial uncoupler.

The application of 1 mM HCQ resulted in a considerable rise in TMRM (+) MFI to 918.8 ± 26.0% and cell population to 99.4 ± 0.1% (p <0.0001). This indicates that HCQ leads to hyperpolarization of the mitochondria and possible malfunction(17). As was mentioned in the preceding section, HCQ treatment resulted in the greatest MFI values for both ROS generation and mitochondrial membrane potential. Consistent with our findings, various studies in the literature indicate that mitochondrial hyperpolarization increases ROS production(18).

Although ZEA (10 μM) administration alone raised MFI values to 152.3±11.9%, the population percentage was constant at 89.1±4.3%, similar to the control, suggesting that ZEA may enhance ΔΨm by integrating into mitochondrial membranes or regulating the ETC(19). This hyperpolarization induces ROS generation in some cells (increased ROS+ cells), while zeaxanthin's antioxidant activities balance this ROS rise across the population, as seen in the previous section.

Cotreatment of 1 mM HCQ and 10 μM ZEA resulted in a markedly enhanced TMRM (+) population of 97.7 ± 2.9% and a significantly high MFI of 705.8 ± 26.3% (p < 0.0001). When analyzed in conjunction with the ROS production results, HCQ+ZEA generated the greatest ROS (+) population (43.5%) and a high TMRM+ population ratio (97.7%). However, the corresponding MFI values were lower compared to HCQ (ROS: 129.9%; TMRM+: 705.8%). These findings suggest that ZEA reduces the density of ROS and ΔΨm in individual cells while increasing the number of cells engaged in ROS production and ΔΨm, modulating the effect of HCQ.

Please insert Figure 5

**3.5 Effect of ZEA on Lysosomal Membrane Permeabilization**

Lysosomal membrane permeabilization (LMP) induces hydrolase leakage into the cytosol, which may inhibit cell function and induce apoptosis or necrosis under physiological or pathological conditions(20). In healthy cells, AO exhibits punctate red fluorescence, whereas the loss of the lysosomal proton gradient enhances cytosolic green fluorescence and decreases red lysosomal signaling. AO staining was employed to measure red (lysosomal) and green (cytosolic) fluorescence signals at 24 and 72 hours in ARPE-19 cells, as shown in Figure 6. The red intensity signal per well was divided by the green intensity for normalization as recommended by Thome et al(21).

At 24 hours, the control group exhibited a mean red/green ratio of 0.0306 ± 0.0004, indicating baseline lysosomal activity. HCQ treatment significantly reduced the ratio to 0.0214 ± 0.0001, consistent with its role as a lysosomal inhibitor(2). Zeaxanthin alone resulted in a ratio of 0.0283 ± 0.0005, which was comparable to the control, indicating minimal impact on basal lysosomal activity. The combination treatment showed the lowest ratio of 0.0186 ± 0.0005, indicating enhanced lysosomal pH neutralization.

At 72 hours, a similar trend was observed, with all groups showing reduced ratios compared to 24 hours, likely reflecting prolonged cellular stress or adaptation. The control group had a mean ratio of 0.0127 ± 0.0013. HCQ treatment further decreased the ratio to 0.0035 ± 0.0002, consistent with sustained lysosomal inhibition. ZEA maintained a ratio of 0.0127 ± 0.0003, similar to the control. Notably, the combination treatment resulted in the lowest ratio of 0.0038 ± 0.0001, confirming a synergistic effect on lysosomal dysfunction.

These findings support HCQ's mechanism of preventing lysosomal acidification by inhibiting vacuolar H⁺-ATPase (V-ATPase), which leads to decreased AO red fluorescence(2). The reduction in red/ green ratio in the HCQ-treated group indicates AO leakage into the cytosol due to LMP or pH neutralization. The lack of a substantial effect on the red/green ratio suggests that ZEA does not significantly modify basal lysosomal pH, despite the observed increase in ROS and MMP in this study. This increase in ROS/MMP may be due to a transient pro-oxidant effect, causing an early adaptive stress response without changing LMP. Carotenoids such as ZEA have particular pro- or antioxidant effects which are determined by dose and cellular environment(15, 16).

The combination treatment exhibited the most pronounced reduction in red/green ratio, indicating a synergistic enhancement of lysosomal pH neutralization. This could result from ZEA's lipophilic nature amplifying HCQ's lysosomal accumulation, disrupting lysosomal integrity(22). This is consistent with the lower ratio of the combination group when compared to HCQ alone treatment, particularly at 24 hours.

Please insert Figure 6

**3.6 Metabolomics Analyses**

**Significantly Altered Metabolites**

Metabolomic analyses showed that HCQ treatment led to significant changes in amino acid metabolism, the TCA cycle, purine catabolism, lipid biosynthesis, and phase II conjugation pathways in ARPE-19 cells, as shown in Figure 7 and supporting Table 1. While ZEA alone treatment had limited effects, the combination group significantly reduced the HCQ-induced metabolic disturbances. The HCQ-treated group showed lower levels of taurine (−54.9), hippuric acid (−19.8), GABA (−9.5), citrate (−3.0), malate (−3.4), hypoxanthine (−3.8), xanthine (−5.6), and uric acid (−11.4). On the other hand, palmitic acid (+87.6), prostaglandin H₂ (+13.9), and S-lactoylglutathione (+12.54) levels were increased. These findings are consistent with decreased mitochondrial energy production, decreased redox buffering capacity, and activation of the inflammatory response. Increases in citrate (+3.16) and pantothenic acid (+3.28) levels were noted in the combination group. These changes suggest that ZEA mitigates HCQ-induced oxidative damage by supporting mitochondrial energy metabolism and glutathione biosynthesis.

Please insert Figure 7

**VIP Score Analysis and Pathway Enrichment**

PLS-DA-based VIP score analysis showed that the metabolites with the highest discriminatory power were 5-aminoimidazole-4-carboxamide (AICA), hypoxanthine, phenylacetylglutamine, phylloquinone, cholestenone, and taurine (VIP > 1.3) as seen in supporting Table 2. These compounds were associated with purine catabolism, vitamin B₆ metabolism, amino acid, and lipid pathways. Pathway enrichment analysis in Figure 8 showed that the lowest p-values were obtained for purine metabolism (p = 1.1×10⁻³, C–HZ), phenylacetate metabolism (p = 1.0×10⁻²), and pantothenate/coenzyme A biosynthesis (p = 5.2×10⁻²). These findings suggest that nucleotide catabolism and energy metabolism in the cell are suppressed by HCQ, and ZEA partially reverses these effects (Supporting Table 3).

Please insert Figure 8

**Metabolite–Functional Correlation Analysis**

Biological data obtained through ANX-AAD7, DCFDA, TMRM, and AO analyses were integrated with metabolomics findings. Pearson correlation analysis results are presented in Figure 9. Taurine, GABA, hypoxanthine, uric acid, and citrate showed strong positive correlations with cell viability and strong negative correlations with ROS and MMP loss (r ≈ ±0.9). In contrast, palmitic acid exhibited positive correlations with ROS and MMP levels, and negative correlations with viability (r = +0.97 and −0.62). This pattern aligns with our results showing that HCQ suppresses autophagy, resulting in lipophagy deficit and an increased oxidative burden. In the presence of ZEA, some of these correlations were weakened, especially the recovery in taurine and citrate levels, indicating a rebalancing of mitochondrial function.

Please insert Figure 9

**4 DISCUSSION**

The present study demonstrates, for the first time to our knowledge, that ZEA exerts a marked protective effect against HCQ-induced cytotoxicity in ARPE-19 cells. HCQ exposure significantly reduced cell viability, increased necrotic cell populations, elevated ROS levels, and disrupted both ΔΨm and lysosomal pH. Combination treatment with ZEA substantially mitigated these deleterious effects, suggesting that its protective activity may be mediated through antioxidant and mitochondrial stabilizing mechanisms.

In this study, HCQ significantly decreased cell viability, aligning with previous studies reporting retinal pigment epithelial degeneration due to lysosomal dysfunction and oxidative stress (1, 23, 24). Notably, ZEA combination treatment restored viability in a dose-dependent manner. This cytoprotective effect is in line with earlier reports on ZEA's ability to enhance cell viability by modulating redox homeostasis and activating endogenous antioxidant defenses (10). However, it should be noted that the aim of our study is not to determine a safe dose threshold that provides complete protection, but rather to reveal the extent to which ZEA can modulate the cellular response under significant HCQ toxicity. Therefore, the results suggest that ZEA does not completely eliminate HCQ toxicity but significantly reduces cellular damage and exhibits a partial cytoprotective effect.

Although the free radical scavenging effect of ZEA was not directly evaluated with chemical tests in our study, the DCFDA and TMRM results show that ZEA has an effect on cellular redox balance and mitochondrial function. DCFDA assay revealed a significant reduction of HCQ-induced ROS by ZEA combination treatment. This finding supports the proposed role of ZEA as a potent singlet oxygen and peroxyl radical quencher in ocular tissues (25). The simultaneous restoration of ΔΨm observed in the TMRM assay further emphasizes the mitochondrial protective role of ZEA. These findings suggest that ZEA can modulate mitochondrial activity and regulate the redox response without causing cytotoxicity under basal conditions. These effects of ZEA likely underlies its cytoprotective activity. In addition to direct free radical scavenging, as suggested in previous studies, ZEA can upregulate the expression of mitochondrial antioxidant enzymes such as superoxide dismutase (SOD2) and glutathione peroxidase (GPx), decline ROS-mediated damage, and stabilize mitochondrial function, as supported by our TMRM data (10). Furthermore, ZEA can integrate into cell membranes, increasing membrane fluidity and resistance to oxidative damage, thereby stabilizing the mitochondrial membrane(26).

Previous studies have demonstrated that HCQ inhibits autophagy by impairing lysosomal acidification and blocking the final step of the autophagic flux, which aligns with our AO staining findings (2). This mechanism leads to the accumulation of damaged mitochondria, mitophagy failure, and oxidative stress. Interestingly, despite enhanced lysosomal pH neutralization, ZEA significantly mitigated HCQ-induced cell death in this study. This protective effect likely stems from ZEA’s antioxidant properties, counteracting HCQ-induced oxidative stress. This modulation may explain how ZEA can both expand lysosomal deacidification and decrease HCQ toxicity.

Our metabolomics analyses demonstrate that redox imbalance, suppression of purine metabolism, and decline in lipophagy are the primary biochemical mechanisms responsible for the retinotoxic effects of HCQ. Dramatic losses of taurine and GABA in amino acid metabolism suggest depletion of critical reserves for retinal osmoregulation and antioxidant defense. Taurine deficiency is known to be associated with photoreceptor degeneration(27, 28). The reduction in these metabolites is directly related to the increase in early apoptosis and necrosis observed in the ANX-AAD7 assay. Therefore, HCQ-induced taurine depletion may be part of the mechanism of retinotoxicity.

In addition, metabolomic analyses indicate that HCQ treatment disrupts mitochondrial-related metabolic pathways. Specifically, the decrease in citrate and malate levels, key metabolites of the TCA cycle, suggests suppression of mitochondrial energy metabolism. Suppression of purine metabolism, particularly the decrease in uric acid levels, led to a decrease in redox buffer capacity. The free radical scavenging properties of uric acid are well demonstrated (29, 30). Therefore, the reduction in uric acid in the HCQ group may have facilitated ROS accumulation and mitochondrial depolarization.

The increase in palmitic acid in lipid metabolism can be explained by the suppression of lipophagy. Lipophagy is a specialized form of autophagy that regulates free fatty acid homeostasis by breaking down lipid droplets. Singh et al. (31) showed that blocking lipophagy leads to free fatty acid accumulation. Increased palmitic acid and positive correlation of ROS are consistent with this mechanism(32). The simultaneous increase in prostaglandins suggests activation of the inflammatory response.

The protective effect of ZEA in the combination group was also evident at the metabolomic levels. The protective effect of ZEA in the combination group was also evident at the metabolomic level. In the combination group, recovery in citrate and pantothenate levels suggests partial restoration of mitochondrial energy metabolism and support of coenzyme A biosynthesis. In parallel, ZEA treatment raised pantothenate, γ-glutamylcysteine, and citrate levels while reducing ROS, partially preserving MMP, and reducing necrosis. Previous reports have demonstrated that increased pantothenate and γ-glutamylcysteine levels promote the reduction of ROS through the Nrf2 pathway(33, 34). Citrate recovery points to improved mitochondrial energy metabolism(35). Increased steroid glucuronides also point to enhanced phase II detoxification capacity and the removal of HCQ-induced toxic metabolites(36). These findings are consistent with reports that zeaxanthin increases phase II antioxidant enzymes by activating the Nrf2 pathway(10, 37).

**5 CONCLUSION**

This study demonstrates that HCQ causes toxicity in retinal cells via autophagy blockade, mitochondrial dysfunction, redox imbalance, and lysosomal neutralization, while ZEA exhibits antioxidant, mitochondrial, and lysosomal protective effects. These findings suggest that zeaxanthin may be considered a potential protective agent against HCQ-induced retinotoxicity.

**Author Contributions**

**Münire Berna Asal Altıparmak:** Biological analyses, methodology, visualization, writing-reviewing and editing,  **Emine Merve Yıldırım:** biological analyses, methodology, visualization, writing-reviewing and editing, **Ozan Kaplan:** metabolomics analyses, methodology, visualization, writing-original draft, reviewing and editing, **Emine Koç:** metabolomics analyses, methodology, visualization, **Açelya Erikçi:** biological analyses, writing-reviewing and editing, **Mustafa Çelebier:** metabolomics analyses, methodology, visualization, writing-original draft, reviewing and editing, **Tuba Tüylü Küçükkılınç:** conceptualization, designing methodology, project administration, supervision, validation, resources, analysis and interpretation, writing original draft, reviewing and editing, **Gülberk Uçar:** analysis and interpretation, writing-reviewing and editing.

**Conflicts of Interest**

The authors declare no conflicts of interest.

**Declaration of Generative AI Use**

During the preparation of this manuscript, the authors utilized generative AI to enhance linguistic clarity, grammar, and readability.

**Data Availability Statement**

The data that support the findings of this study are available from the corresponding authors upon reasonable request.

**REFERENCES**

1. Marmor MF, Kellner U, Lai TY, Melles RB, Mieler WF. Recommendations on Screening for Chloroquine and Hydroxychloroquine Retinopathy (2016 Revision). Ophthalmology. 2016;123(6):1386-94.

2. Mauthe M, Orhon I, Rocchi C, Zhou X, Luhr M, Hijlkema KJ, et al. Chloroquine inhibits autophagic flux by decreasing autophagosome-lysosome fusion. Autophagy. 2018;14(8):1435-55.

3. Dunn KC, Aotaki-Keen AE, Putkey FR, Hjelmeland LM. ARPE-19, a human retinal pigment epithelial cell line with differentiated properties. Exp Eye Res. 1996;62(2):155-69.

4. Lahiri V, Hawkins WD, Klionsky DJ. Watch What You (Self-) Eat: Autophagic Mechanisms that Modulate Metabolism. Cell Metabolism. 2019;29(4):803-26.

5. Redmann M, Benavides GA, Berryhill TF, Wani WY, Ouyang X, Johnson MS, et al. Inhibition of autophagy with bafilomycin and chloroquine decreases mitochondrial quality and bioenergetic function in primary neurons. Redox Biology. 2017;11:73-81.

6. Li J-H, Xu Z-Y, Li M-J, Zheng W-L, Huang X-M, Xiao F, et al. LC-MS based metabolomics reveals metabolic pathway disturbance in retinal pigment epithelial cells exposed to hydroxychloroquine. Chemico-Biological Interactions. 2020;328:109212.

7. Joshi P, Dhaneshwar SS. An update on disease modifying antirheumatic drugs. Inflamm Allergy Drug Targets. 2014;13(4):249-61.

8. Olsen NJ, Schleich MA, Karp DR. Multifaceted effects of hydroxychloroquine in human disease. Seminars in Arthritis and Rheumatism. 2013;43(2):264-72.

9. Li B, George EW, Rognon GT, Gorusupudi A, Ranganathan A, Chang F-Y, et al. Imaging lutein and zeaxanthin in the human retina with confocal resonance Raman microscopy. Proceedings of the National Academy of Sciences. 2020;117(22):12352-8.

10. Zou X, Gao J, Zheng Y, Wang X, Chen C, Cao K, et al. Zeaxanthin induces Nrf2-mediated phase II enzymes in protection of cell death. Cell Death Dis. 2014;5(5):e1218.

11. Eriksson I, Vainikka L, Persson HL, Öllinger K. Real-Time Monitoring of Lysosomal Membrane Permeabilization Using Acridine Orange. Methods Protoc. 2023;6(4).

12. SenthilKumar G, Skiba JH, Kimple RJ. High-Throughput Quantitative Detection of Basal Autophagy and Autophagic Flux Using Image Cytometry. BioTechniques. 2019;67(2):70-3.

13. Kaplan O, Koç E, Türk S, Küçükkılınç TT, Göktaş Z, Çelebier M. Metabolomic signature in ocular dosing: Exploring the metabolic impacts of sublethal high-dose naringenin on ARPE-19 cells. European Journal of Integrative Medicine. 2024;72:102414.

14. Chang K-C, Liu P-F, Chang C-H, Lin Y-C, Chen Y-J, Shu C-W. The interplay of autophagy and oxidative stress in the pathogenesis and therapy of retinal degenerative diseases. Cell & Bioscience. 2022;12(1):1.

15. Young AJ, Lowe GM. Antioxidant and Prooxidant Properties of Carotenoids. Archives of Biochemistry and Biophysics. 2001;385(1):20-7.

16. Ribeiro D, Freitas M, Silva AMS, Carvalho F, Fernandes E. Antioxidant and pro-oxidant activities of carotenoids and their oxidation products. Food and Chemical Toxicology. 2018;120:681-99.

17. Seydi E, Hassani MK, Naderpour S, Arjmand A, Pourahmad J. Cardiotoxicity of chloroquine and hydroxychloroquine through mitochondrial pathway. BMC Pharmacology and Toxicology. 2023;24(1):26.

18. Zorov DB, Juhaszova M, Sollott SJ. Mitochondrial ROS-induced ROS release: An update and review. Biochimica et Biophysica Acta (BBA) - Bioenergetics. 2006;1757(5):509-17.

19. Kupisz K, Sujak A, Patyra M, Trebacz K, Gruszecki WI. Can membrane-bound carotenoid pigment zeaxanthin carry out a transmembrane proton transfer? Biochimica et Biophysica Acta (BBA) - Biomembranes. 2008;1778(10):2334-40.

20. Repnik U, Hafner Česen M, Turk B. Lysosomal membrane permeabilization in cell death: Concepts and challenges. Mitochondrion. 2014;19:49-57.

21. Thomé MP, Filippi-Chiela EC, Villodre ES, Migliavaca CB, Onzi GR, Felipe KB, et al. Ratiometric analysis of Acridine Orange staining in the study of acidic organelles and autophagy. J Cell Sci. 2016;129(24):4622-32.

22. Kornhuber J, Henkel AW, Groemer TW, Städtler S, Welzel O, Tripal P, et al. Lipophilic cationic drugs increase the permeability of lysosomal membranes in a cell culture system. J Cell Physiol. 2010;224(1):152-64.

23. Yusuf IH, Charbel Issa P, Ahn SJ. Hydroxychloroquine-induced Retinal Toxicity. Front Pharmacol. 2023;14:1196783.

24. Dhillon B, Singh S, Keifer J, Kumar U, Shaikh S, Ho S, et al. Ameliorating hydroxychloroquine induced retinal toxicity through cerium oxide nanoparticle treatments. J Biomater Appl. 2022;36(6):1033-41.

25. Roberts JE, Dennison J. The Photobiology of Lutein and Zeaxanthin in the Eye. Journal of Ophthalmology. 2015;2015(1):687173.

26. Sujak A, Gabrielska J, Grudziński W, Borc R, Mazurek P, Gruszecki WI. Lutein and Zeaxanthin as Protectors of Lipid Membranes against Oxidative Damage: The Structural Aspects. Archives of Biochemistry and Biophysics. 1999;371(2):301-7.

27. Ripps H, Shen W. Review: taurine: a "very essential" amino acid. Mol Vis. 2012;18:2673-86.

28. Fan Y, Lai J, Yuan Y, Wang L, Wang Q, Yuan F. Taurine Protects Retinal Cells and Improves Synaptic Connections in Early Diabetic Rats. Current Eye Research. 2020;45(1):52-63.

29. Glantzounis GK, Tsimoyiannis EC, Kappas AM, Galaris DA. Uric Acid and Oxidative Stress. Current Pharmaceutical Design. 2005;11(32):4145-51.

30. Duarte AI, Santos MS, Oliveira CR, Rego AC. Insulin neuroprotection against oxidative stress in cortical neurons—Involvement of uric acid and glutathione antioxidant defenses. Free Radical Biology and Medicine. 2005;39(7):876-89.

31. Singh R, Kaushik S, Wang Y, Xiang Y, Novak I, Komatsu M, et al. Autophagy regulates lipid metabolism. Nature. 2009;458(7242):1131-5.

32. Khawar MB, Gao H, Li W. Autophagy and Lipid Metabolism. In: Qin Z-H, editor. Autophagy: Biology and Diseases: Basic Science. Singapore: Springer Singapore; 2019. p. 359-74.

33. Slyshenkov VS, Dymkowska D, Wojtczak L. Pantothenic acid and pantothenol increase biosynthesis of glutathione by boosting cell energetics. FEBS Lett. 2004;569(1-3):169-72.

34. Ma Q. Role of nrf2 in oxidative stress and toxicity. Annu Rev Pharmacol Toxicol. 2013;53:401-26.

35. Iacobazzi V, Infantino V. Citrate – new functions for an old metabolite. Biological Chemistry. 2014;395(4):387-99.

36. Al-Ghananeem AM, Crooks PA. Phase I and Phase II Ocular Metabolic Activities and the Role of Metabolism in Ophthalmic Prodrug and Codrug Design and Delivery. Molecules. 2007;12(3):373-88.

37. Ying C, Chen L, Wang S, Mao Y, Ling H, Li W, et al. Zeaxanthin ameliorates high glucose-induced mesangial cell apoptosis through inhibiting oxidative stress via activating AKT signalling-pathway. Biomedicine & Pharmacotherapy. 2017;90:796-805.

**Tables**

**Supporting Table 1.** Fold change values of control (C), hydroxychloroquine (H), zeaxanthin (Z), and combination (HZ) groups. Positive values indicate upregulation and negative values indicate downregulation relative to the reference group.

|  | **Fold Change** | | | | | |
| --- | --- | --- | --- | --- | --- | --- |
|  | **H/C** | **HZ/C** | **Z/C** | **HZ/H** | **HZ/Z** | **H/Z** |
| gamma-Aminobutyric acid | -9.49 | -8.85 | -1.50 | 1.07 | -5.90 | -6.32 |
| Methylarsonate | -3.20 | -3.13 | -4.64 | 1.02 | 1.48 | 1.45 |
| 4-Hydroxybenzaldehyde | -2.15 | -1.43 | 1.14 | 1.50 | -1.63 | -2.46 |
| Taurine | -54.96 | -8.22 | -1.39 | 6.69 | -5.89 | -39.40 |
| Pyroglutamic acid | -1.10 | 1.07 | -1.41 | 1.18 | 1.50 | 1.28 |
| D-Malic acid | -3.43 | -3.02 | -1.70 | 1.13 | -1.78 | -2.02 |
| Hypoxanthine | -3.79 | -3.75 | -1.26 | 1.01 | -2.97 | -3.00 |
| Glutamine | -1.89 | -1.75 | -1.61 | 1.08 | -1.09 | -1.17 |
| Xanthine | -5.60 | -6.21 | -2.68 | -1.11 | -2.31 | -2.08 |
| D-Ribose | -1.70 | -1.55 | 1.43 | 1.09 | -2.23 | -2.43 |
| Uric acid | -11.42 | -6.73 | -2.91 | 1.70 | -2.31 | -3.92 |
| N-Acetyl-L-aspartic acid | -1.99 | 1.09 | 1.10 | 2.16 | -1.01 | -2.19 |
| Oxalosuccinic acid | 1.14 | 1.11 | -1.39 | -1.02 | 1.55 | 1.58 |
| Hippuric acid | -19.85 | -14.19 | -4.46 | 1.40 | -3.18 | -4.45 |
| 3-Carbamoyl-2-phenylpropionaldehyde | -1.38 | -1.17 | -1.20 | 1.18 | 1.03 | -1.14 |
| Citric acid | -3.03 | 1.05 | -2.99 | 3.16 | 3.12 | -1.01 |
| 4-Pyridoxic acid | -2.33 | -1.50 | -1.82 | 1.56 | 1.22 | -1.28 |
| 5-Aminoimidazole-4-carboxamide | -23.32 | -16.58 | -28.62 | 1.41 | 1.73 | 1.23 |
| 3,4-Dihydroxyphenylglycol | -1.39 | -1.61 | -1.39 | -1.16 | -1.16 | 1.00 |
| D-Fructose | -1.19 | -1.45 | 1.71 | -1.22 | -2.48 | -2.04 |
| 1,2-Dihydronaphthalene-1,2-diol | -1.53 | -1.40 | -1.35 | 1.09 | -1.04 | -1.14 |
| Pantothenic acid | 1.90 | 3.28 | 1.17 | 1.73 | 2.80 | 1.62 |
| Palmitic acid | 87.58 | 67.17 | 70.04 | -1.30 | -1.04 | 1.25 |
| Phenylacetylglutamine | -15.28 | -16.33 | -5.11 | -1.07 | -3.20 | -2.99 |
| 13-OxoODE | -2.23 | 1.90 | -2.44 | 4.24 | 4.63 | 1.09 |
| gamma-Glutamylcysteine | 1.27 | 1.12 | 1.19 | -1.13 | -1.07 | 1.06 |
| 15-HETE | -1.01 | 1.95 | 3.39 | 1.98 | -1.74 | -3.44 |
| Pantetheine | -2.07 | -1.85 | -1.34 | 1.12 | -1.38 | -1.55 |
| Prostaglandin B1 | 6.12 | 2.49 | 1.25 | -2.46 | 1.99 | 4.91 |
| Prostaglandin H2 | 13.99 | 3.10 | 1.40 | -4.52 | 2.21 | 10.00 |
| 6-Methylthiopurine 5'-monophosphate ribonucleotide | 1.05 | 1.27 | -1.49 | 1.22 | 1.90 | 1.56 |
| S-Lactoylglutathione | 12.54 | 22.45 | 7.33 | 1.79 | 3.06 | 1.71 |
| Farnesyl pyrophosphate | 7.41 | 41.99 | 3.70 | 5.67 | 11.35 | 2.00 |
| Cholestenone | -28.18 | -16.52 | -3.93 | 1.71 | -4.20 | -7.17 |
| Phylloquinone | -26.04 | -38.52 | -17.53 | -1.48 | -2.20 | -1.49 |
| Dolichyl b-D-glucosyl phosphate | -2.45 | 1.42 | -3.41 | 3.48 | 4.85 | 1.39 |
| 2-Methoxy-estradiol-17b 3-glucuronide | -7.33 | 2.21 | -5.27 | 16.23 | 11.66 | -1.39 |
| dUDP | -1.38 | -1.36 | -1.66 | 1.02 | 1.23 | 1.20 |
| 2-Methoxyestrone 3-glucuronide | -5.52 | 1.53 | -2.11 | 8.44 | 3.22 | -2.62 |
| Glycocholic acid | 5.42 | 1.38 | 1.12 | -3.94 | 1.22 | 4.83 |
| Coprocholic acid | -1.60 | -1.12 | 1.99 | 1.42 | -2.24 | -3.18 |
| Chlordecone alcohol | 1.48 | 1.38 | -1.31 | -1.08 | 1.81 | 1.95 |
| Inosine triphosphate | -1.10 | -1.12 | 1.06 | -1.02 | -1.20 | -1.17 |
| 3-Oxotetradecanoyl-CoA | 1.34 | 1.29 | 1.12 | -1.04 | 1.15 | 1.19 |

**Supporting Table 2.** VIP (Variable Importance in Projection) scores from PLS-DA analysis. Component 1 and Component 2 show the contribution of each metabolite to the first and second latent variables of the PLS-DA model, respectively. Metabolites with VIP scores > 1.3 are considered highly important for group discrimination.

| **Metabolite** | **Component 1** | **Component 2** |
| --- | --- | --- |
| 5-Aminoimidazole-4-carboxamide | 1.78 | 1.54 |
| dUDP | 1.70 | 1.47 |
| Phenylacetylglutamine | 1.58 | 1.38 |
| Methylarsonate | 1.57 | 1.36 |
| Hippuric acid | 1.50 | 1.32 |
| Phylloquinone | 1.48 | 1.30 |
| Glutamine | 1.43 | 1.27 |
| 15-HETE | 1.41 | 1.34 |
| Xanthine | 1.39 | 1.23 |
| Coprocholic acid | 1.38 | 1.46 |
| Cholestenone | 1.31 | 1.16 |
| Uric acid | 1.31 | 1.18 |
| Pyroglutamic acid | 1.20 | 1.07 |
| 4-Pyridoxic acid | 1.14 | 1.06 |
| Oxalosuccinic acid | 1.12 | 1.18 |
| 1,2-Dihydronaphthalene-1,2-diol | 1.12 | 1.03 |
| 3,4-Dihydroxyphenylglycol | 1.10 | 0.95 |
| D-Malic acid | 1.05 | 1.01 |
| Palmitic acid | 0.95 | 0.90 |
| Citric acid | 0.93 | 0.85 |
| 3-Carbamoyl-2-phenylpropionaldehyde | 0.87 | 0.94 |
| Pantetheine | 0.81 | 0.89 |
| N-Acetyl-L-aspartic acid | 0.77 | 1.12 |
| S-Lactoylglutathione | 0.75 | 0.67 |
| 6-Methylthiopurine 5'-monophosphate ribonucleotide | 0.73 | 0.77 |
| D-Ribose | 0.72 | 0.97 |
| gamma-Glutamylcysteine | 0.71 | 0.68 |
| Chlordecone alcohol | 0.66 | 1.02 |
| gamma-Aminobutyric acid | 0.64 | 0.80 |
| D-Fructose | 0.63 | 0.77 |
| Dolichyl b-D-glucosyl phosphate | 0.61 | 0.55 |
| Farnesyl pyrophosphate | 0.60 | 0.53 |
| Inosine triphosphate | 0.53 | 0.87 |
| Pantothenic acid | 0.52 | 0.47 |
| 4-Hydroxybenzaldehyde | 0.50 | 0.89 |
| 3-Oxotetradecanoyl-CoA | 0.48 | 0.61 |
| Taurine | 0.46 | 0.79 |
| Hypoxanthine | 0.42 | 0.67 |
| Prostaglandin H2 | 0.41 | 0.96 |
| Glycocholic acid | 0.35 | 0.89 |
| Prostaglandin B1 | 0.29 | 0.91 |
| 13-OxoODE | 0.13 | 0.26 |
| 2-Methoxy-estradiol-17b 3-glucuronide | 0.10 | 0.28 |
| 2-Methoxyestrone 3-glucuronide | 0.10 | 0.47 |

**Supporting Table 3.** Pathway enrichment *p* value*s* of control (C), hydroxychloroquine (H), zeaxanthin (Z), and combination (HZ) groups (e.g., C_H = Control vs HCQ; HZ_H = Combination vs HCQ).

|  | ***p* value** | | | | | |
| --- | --- | --- | --- | --- | --- | --- |
| **Pathway** | **C_H** | **C_HZ** | **C_Z** | **H_Z** | **HZ_H** | **HZ_Z** |
| Purine Metabolism | 4.54E-03 | 1.12E-03 | 9.29E-03 | 9.29E-03 | - | 5.15E-02 |
| Phenylacetate Metabolism | 1.65E-02 | 1.02E-02 | 1.13E-02 | 1.13E-02 | - | 1.67E-01 |
| Glutamate Metabolism | 9.32E-02 | 2.12E-01 | 5.83E-02 | 2.30E-01 | - | 6.29E-01 |
| Bile Acid Biosynthesis | 1.83E-01 | 7.04E-01 | 1.20E-01 | 1.20E-01 | 3.76E-02 | 1.35E-01 |
| Aspartate Metabolism | 1.90E-01 | 4.76E-01 | 4.94E-01 | 1.40E-01 | 3.49E-01 | 5.02E-01 |
| Fatty Acid Elongation In Mitochondria | 1.90E-01 | 4.76E-01 | 4.94E-01 | 4.94E-01 | 3.49E-01 | 1.52E-01 |
| Pyruvaldehyde Degradation | 2.08E-01 | 1.66E-01 | - | - | 1.14E-01 | 1.83E-01 |
| Taurine and Hypotaurine Metabolism | 2.44E-01 | 1.96E-01 | 2.06E-01 | 2.06E-01 | 1.35E-01 | 2.16E-01 |
| Fatty acid Metabolism | 2.59E-01 | 5.49E-01 | 5.69E-01 | 5.69E-01 | 4.11E-01 | 2.10E-01 |
| Vitamin B6 Metabolism | 3.59E-01 | 2.94E-01 | 3.07E-01 | - | 2.06E-01 | - |
| Glutathione Metabolism | 3.74E-01 | - | 3.21E-01 | 3.21E-01 | 2.16E-01 | 3.34E-01 |
| Pyrimidine Metabolism | 3.80E-01 | 2.73E-01 | 2.95E-01 | 6.75E-01 | - | 6.94E-01 |
| Warburg Effect | 3.80E-01 | 6.55E-01 | 2.95E-01 | 6.75E-01 | 5.07E-01 | 6.94E-01 |
| Pantothenate and CoA Biosynthesis | 3.89E-01 | 5.23E-02 | 3.34E-01 | 5.77E-02 | 2.26E-01 | 6.34E-02 |
| Transfer of Acetyl Groups into Mitochondria | 4.03E-01 | - | 3.47E-01 | - | 2.35E-01 | 3.61E-01 |
| Estrone Metabolism | 4.31E-01 | 3.56E-01 | 3.72E-01 | - | 2.54E-01 | 3.87E-01 |
| Glycerolipid Metabolism | 4.44E-01 | - | 3.84E-01 | - | 2.63E-01 | 4.00E-01 |
| Cysteine Metabolism | 4.57E-01 | - | 3.96E-01 | - | - | - |
| Mitochondrial Beta-Oxidation of Long Chain Saturated Fatty Acids | 4.83E-01 | 4.02E-01 | - | 4.19E-01 | - | 4.36E-01 |
| Urea Cycle | 4.83E-01 | 4.02E-01 | 4.19E-01 | 4.19E-01 | - | - |
| Pentose Phosphate Pathway | 4.95E-01 | - | - | 4.31E-01 | - | 4.47E-01 |
| Ammonia Recycling | 5.19E-01 | 4.35E-01 | 4.53E-01 | 4.53E-01 | - | - |
| Porphyrin Metabolism | 5.23E-01 | - | - | - | 3.88E-01 | 5.61E-01 |
| Citric Acid Cycle | 5.30E-01 | - | 4.63E-01 | - | 3.24E-01 | 4.81E-01 |
| Propanoate Metabolism | 5.40E-01 | - | 5.60E-01 | 5.60E-01 | - | 5.79E-01 |
| Amino Sugar Metabolism | 5.41E-01 | 4.56E-01 | 1.27E-01 | 1.27E-01 | - | 4.92E-01 |
| Beta-Alanine Metabolism | 5.52E-01 | 4.66E-01 | - | 4.84E-01 | 3.41E-01 | - |
| Fatty Acid Biosynthesis | 5.63E-01 | - | 4.94E-01 | - | 3.49E-01 | 5.12E-01 |
| Nicotinate and Nicotinamide Metabolism | 5.63E-01 | 4.76E-01 | 4.94E-01 | 4.94E-01 | - | - |
| Pyruvate Metabolism | 6.73E-01 | 5.82E-01 | - | - | 4.40E-01 | 6.21E-01 |
| Steroid Biosynthesis | 6.81E-01 | 5.90E-01 | 6.10E-01 | - | 1.09E-01 | 2.48E-01 |
| Arachidonic Acid Metabolism | 8.00E-01 | - | 7.35E-01 | 3.67E-01 | - | - |
| Tyrosine Metabolism | 8.15E-01 | 7.32E-01 | 7.51E-01 | - | - | - |
| Fructose and Mannose Degradation | - | - | 4.53E-01 | 4.53E-01 | - | 4.70E-01 |
| Galactose Metabolism | - | - | 5.24E-01 | 5.24E-01 | - | 5.42E-01 |
| Starch and Sucrose Metabolism | - | - | 4.53E-01 | 4.53E-01 | - | 4.70E-01 |

### Figure Legends

**Figure 1.** Effect of HCQ, ZEA and combination treatment on ARPE-19 cell viability. HCQ (1 mm) significantly reduced cell viability, while ZEA administered in the 1–10 µm range attenuated this toxicity in a dose-dependent manner. Data are shown as mean ± SE (n=3); ***p < 0.001, ****p < 0.0001, compared to the control group.

**Figure 2.** Evaluation of HCQ-induced cell death mechanisms and the protective effect of ZEA using Annexin V/7-AAD flow cytometry. While high viability was observed in the control group, HCQ administration caused significant necrosis. ZEA reduced HCQ-induced necrosis and increased cell viability. Representative flow cytometry diagrams (A–D) and quantitative analysis (E) are provided. Data are shown as mean ± SE (n=3); ****p < 0.0001, compared to the control group.

**Figure 3.** Effect of HCQ, ZEA and combination treatments ROS production in ARPE-19 cells (DCFDA analysis). HCQ significantly increased the percentage of ROS-positive cells, while ZEA alone showed minimal change. The HCQ+ZEA combination partially reduced ROS intensity but increased the percentage of positive cells. Data are shown as mean ± SE (n=3); ***p < 0.001, ****p < 0.0001, compared to the control group.

**Figure 4**. DCFDA fluorescence microscopy images (ROS analysis) of ARPE-19 cells. Low green fluorescence intensity was observed in the control group and significantly higher in the HCQ group. While ZEA alone induced a minimal oxidative response, the ROS distribution appeared heterogeneous among cells in the HCQ+ZEA group. Scale bar = 100 µm.

**Figure 5.** Effect of HCQ, ZEA and their combination on ΔΨm (TMRM analysis). HCQ caused mitochondrial hyperpolarization; ZEA alone maintained ΔΨm values close to control levels. In the combination group the proportion of TMRM (+) cells was increased while MFI was decreased. Representative histograms (A–D) and quantitative data (E) are presented. Data are shown as mean ± SE (n=3); ***p < 0.001, ****p < 0.0001, compared to the control group.

**Figure 6.** LMP determined by AO staining at 24 and 72 hours. HCQ reduced lysosomal acidification, decreasing the red/green ratio. ZEA did not change this parameter, but the HCQ+ZEA combination increased lysosomal pH neutralization. Representative fluorescent images and quantitative data are shown. Data are shown as mean ± SE (n=3); ****p < 0.0001, compared to the control group.

**Figure 7.** Heatmap analysis of metabolites significantly changing in HCQ, ZEA and combination groups in metabolomics analysis

**Figure 8.** PLS-DA VIP scores and pathway enrichment analysis. Purine metabolism, phenylacetate metabolism, and pantothenate/coenzyme A biosynthesis were identified as the most significantly affected pathways (p < 0.05).

**Figure 9.** Pearson correlation analysis between functional parameters (viability, ROS, ΔΨm, AO ratio) and metabolite levels. Taurine, GABA, hypoxanthine, uric acid, and citrate were positively correlated with viability and negatively correlated with ROS and ΔΨm loss. Palmitic acid was positively correlated with ROS.

### Supporting Figure S1. Dose-dependent effect of HCQ on ARPE-19 cell viability after 72 hours of treatment. Cells were exposed to various concentrations of HCQ (15.6–1000 µM), and viability was assessed by MTT assay. Data are presented as mean ± SE (n=3).

### Figures

### Figure 1.


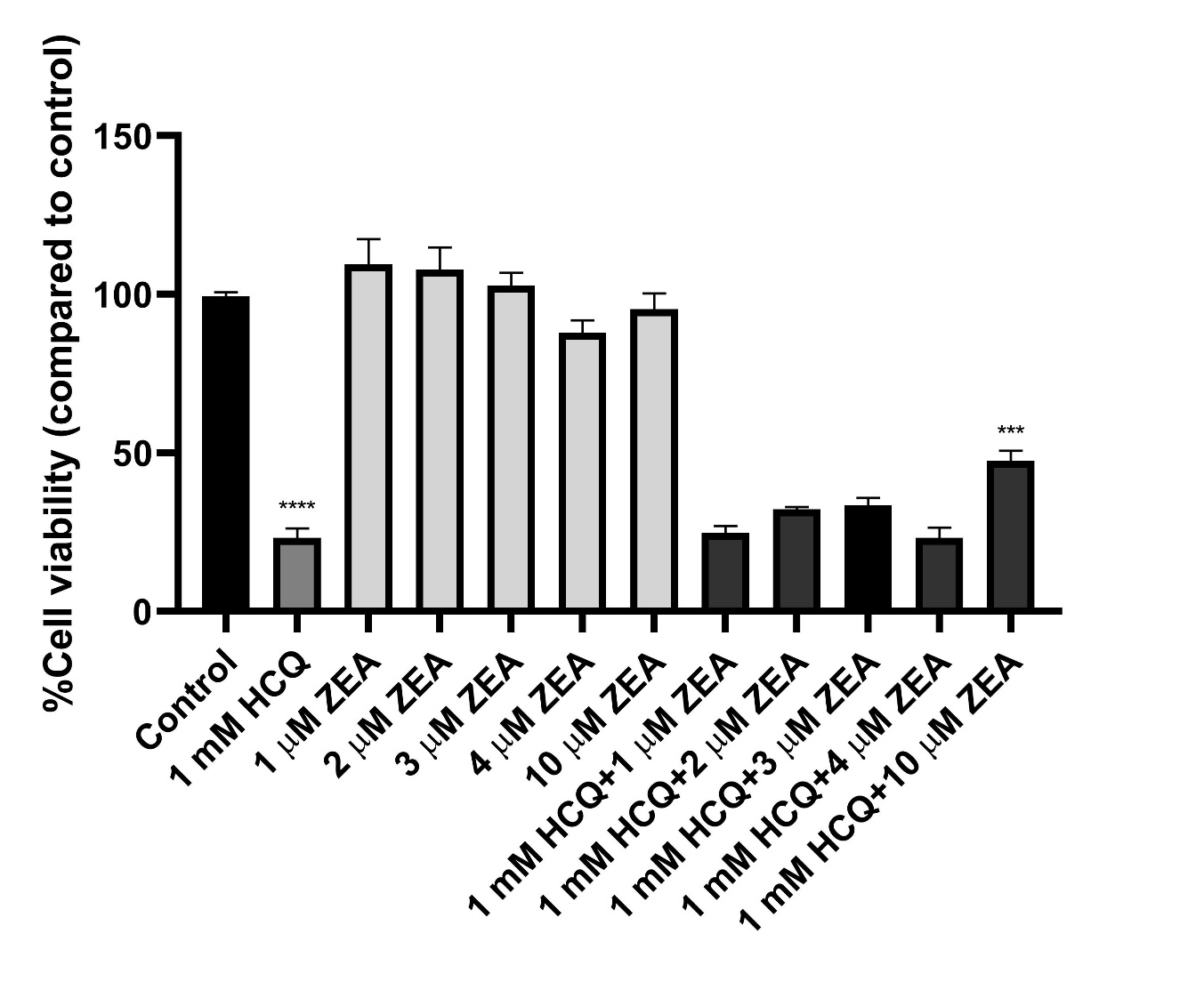


**Figure 1.** Effect of HCQ, ZEA and combination treatment on ARPE-19 cell viability. HCQ (1 mm) significantly reduced cell viability, while ZEA administered in the 1–10 µm range attenuated this toxicity in a dose-dependent manner. Data are shown as mean ± SE (n=3); ***p < 0.001, ****p < 0.0001, compared to the control group.

### Figure 2.

| **A**  **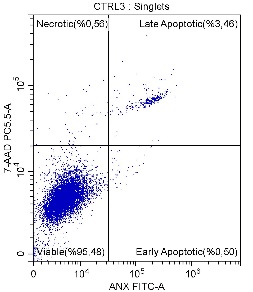** | | **B**  **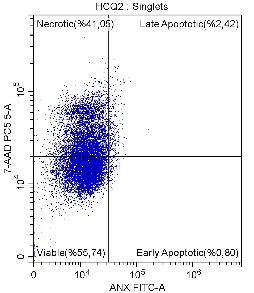** | **E**  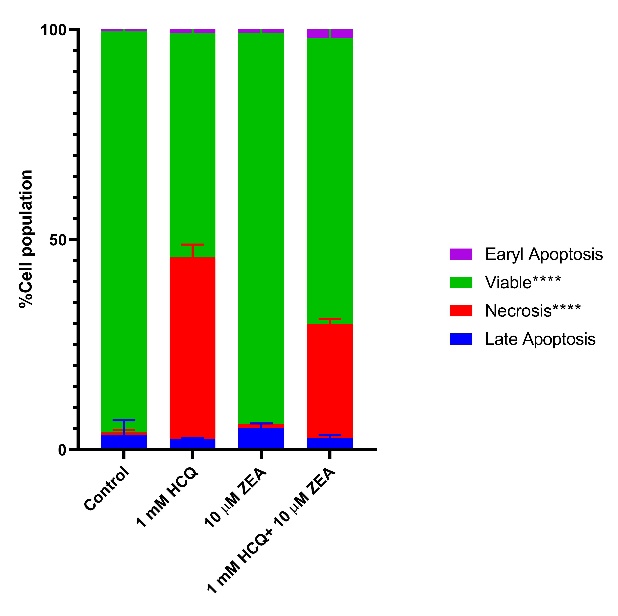 |
| --- | --- | --- | --- |
| **C**  **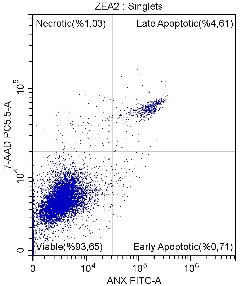** | **D**  **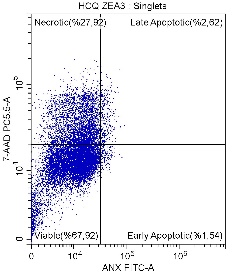** | |  |

**Figure 2.** Evaluation of HCQ-induced cell death mechanisms and the protective effect of ZEA using Annexin V/7-AAD flow cytometry. While high viability was observed in the control group, HCQ administration caused significant necrosis. ZEA reduced HCQ-induced necrosis and increased cell viability. Representative flow cytometry diagrams (A–D) and quantitative analysis (E) are provided. Data are shown as mean ± SE (n=3); ****p < 0.0001, compared to the control group.

### Figure 3.

| **A**  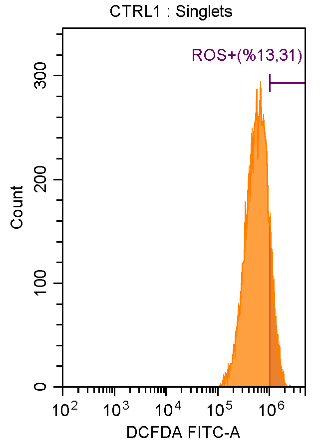 | **B**  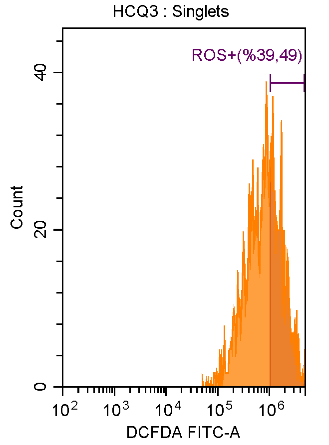 | **E**  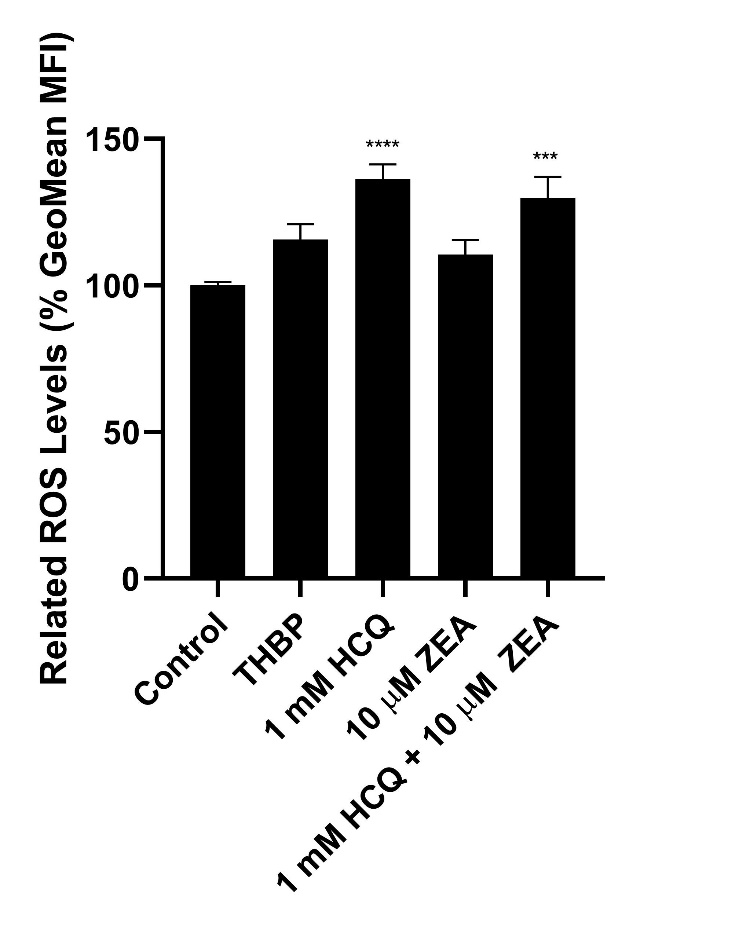 |
| --- | --- | --- |
| **C**  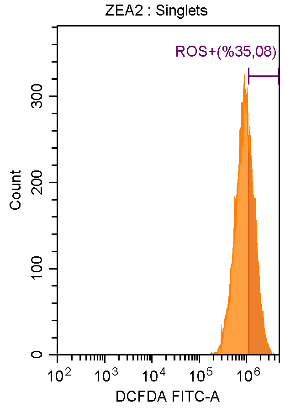 | **D**  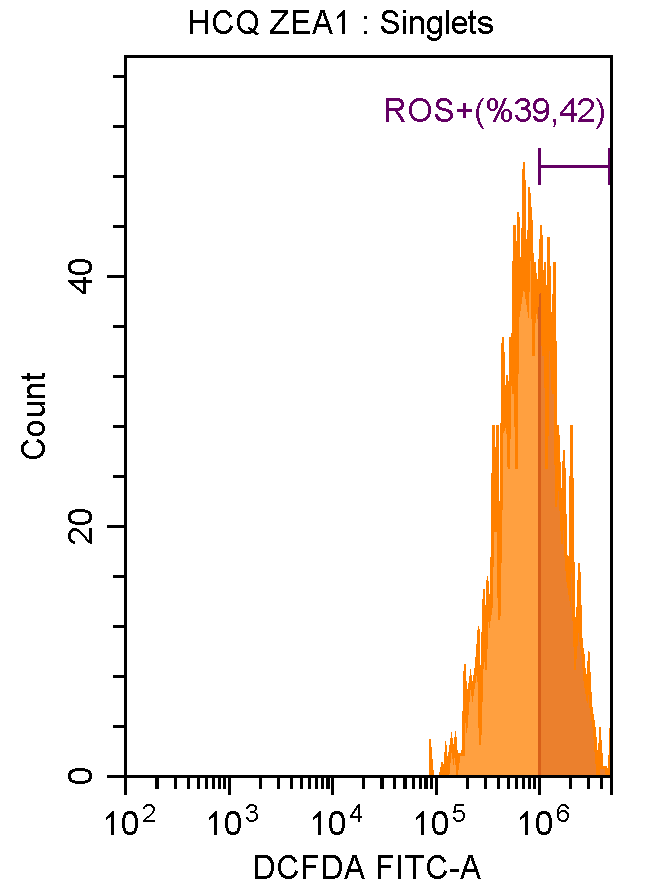 |  |

**Figure 3.** Effect of HCQ, ZEA and combination treatments ROS production in ARPE-19 cells (DCFDA analysis). HCQ significantly increased the percentage of ROS-positive cells, while ZEA alone showed minimal change. The HCQ+ZEA combination partially reduced ROS intensity but increased the percentage of positive cells. Data are shown as mean ± SE (n=3); ***p < 0.001, ****p < 0.0001, compared to the control group.

### Figure 4.

| Control  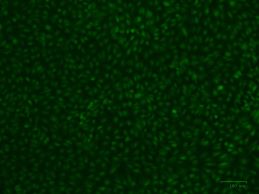 | HCQ  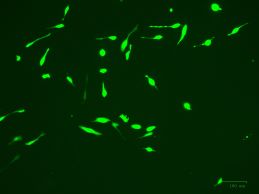 |
| --- | --- |
| ZEA  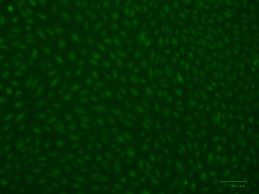 | HCQ+ZEA  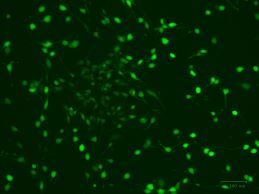 |

**Figure 4**. DCFDA fluorescence microscopy images (ROS analysis) of ARPE-19 cells. Low green fluorescence intensity was observed in the control group and significantly higher in the HCQ group. While ZEA alone induced a minimal oxidative response, the ROS distribution appeared heterogeneous among cells in the HCQ+ZEA group. Scale bar = 100 µm.

**Figure 5**

| **A**  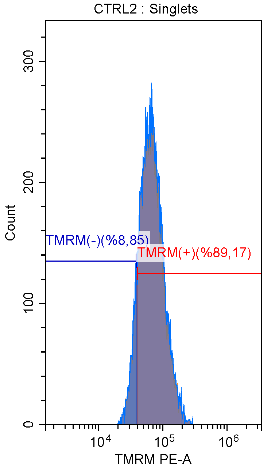 | **B**  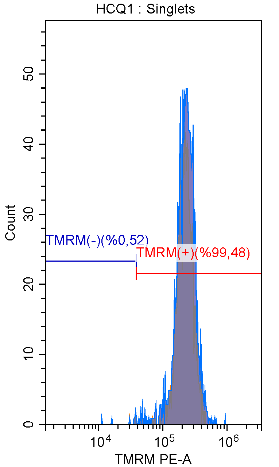 | **E**  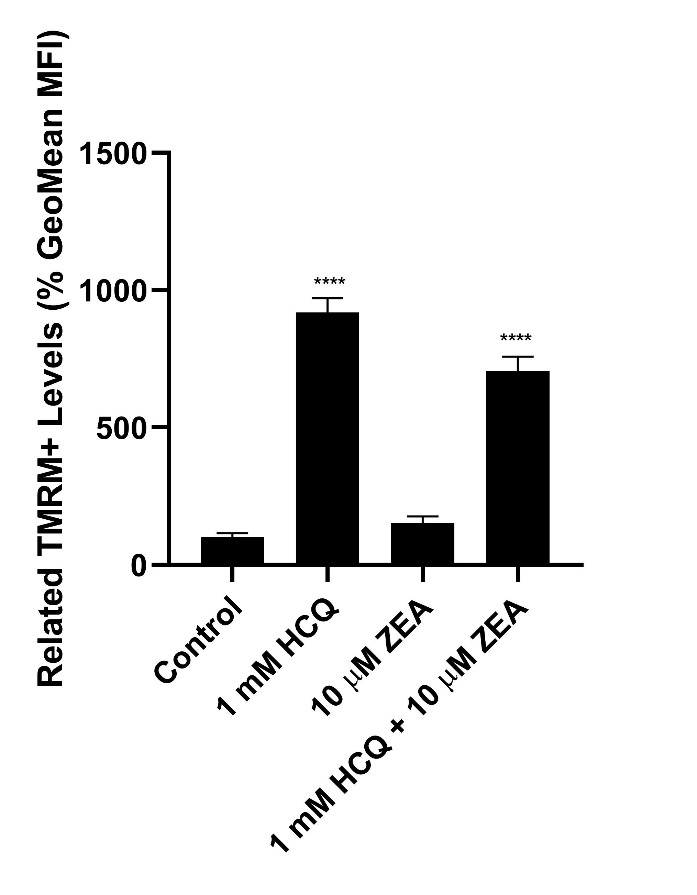 |
| --- | --- | --- |
| **C**  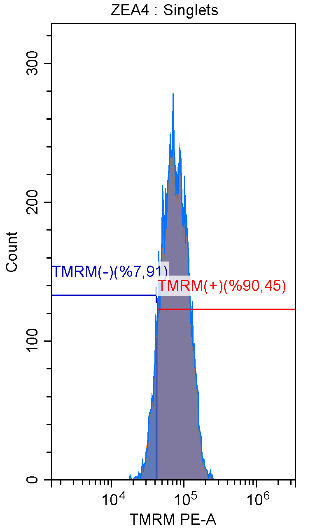 | **D**  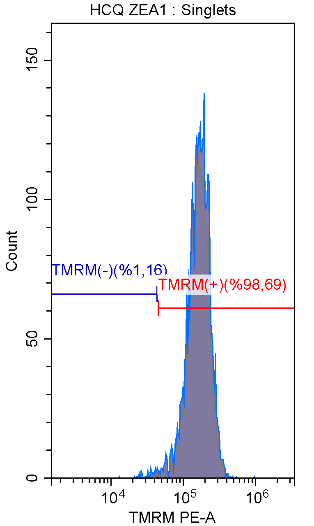 |  |

**Figure 5.** Effect of HCQ, ZEA and their combination on ΔΨm (TMRM analysis). HCQ caused mitochondrial hyperpolarization; ZEA alone maintained ΔΨm values close to control levels. In the combination group the proportion of TMRM (+) cells was increased while MFI was decreased. Representative histograms (A–D) and quantitative data (E) are presented. Data are shown as mean ± SE (n=3); ***p < 0.001, ****p < 0.0001, compared to the control group.

### Figure 6.

| \| C  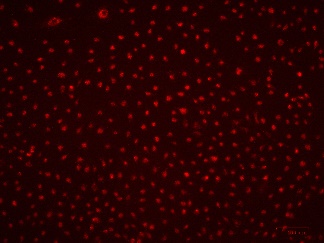 \| 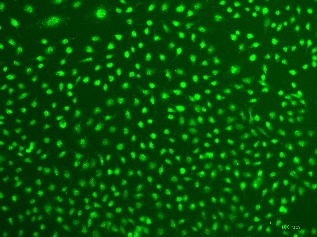 \| HCQ  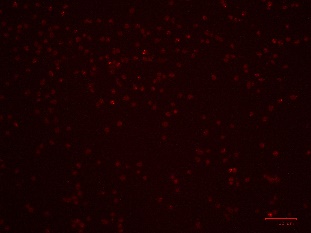 \| 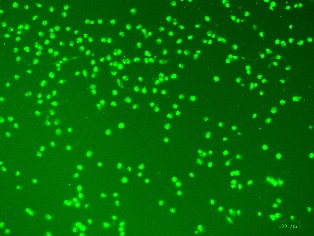 \| \| --- \| --- \| --- \| --- \| \| ZEA  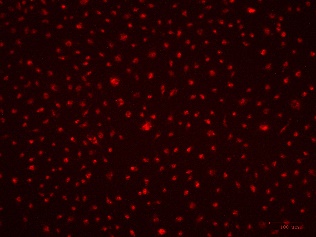 \| 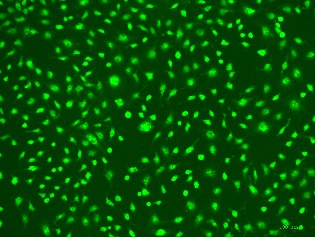 \| HCQ+ZEA  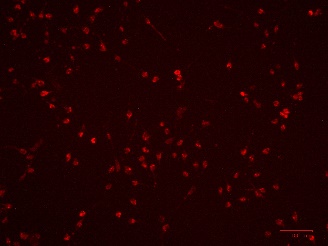 \| 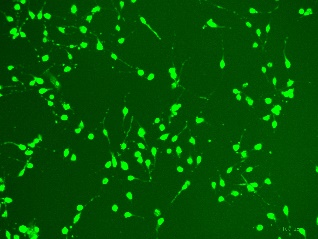 \| \| 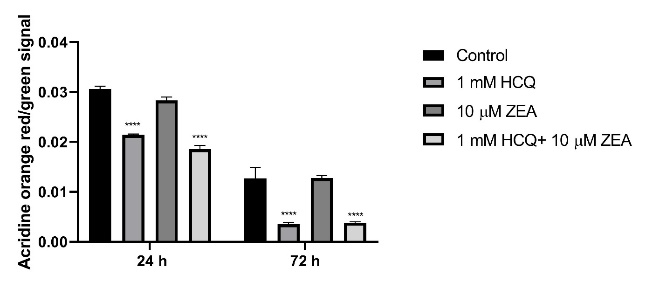 \| \|  \| \| |  |
| --- | --- | --- | --- | --- | --- | --- | --- | --- | --- | --- | --- | --- | --- |

**Figure 6.** LMP determined by AO staining at 24 and 72 hours. HCQ reduced lysosomal acidification, decreasing the red/green ratio. ZEA did not change this parameter, but the HCQ+ZEA combination increased lysosomal pH neutralization. Representative fluorescent images and quantitative data are shown. Data are shown as mean ± SE (n=3); ****p < 0.0001, compared to the control group.

### Figure 7


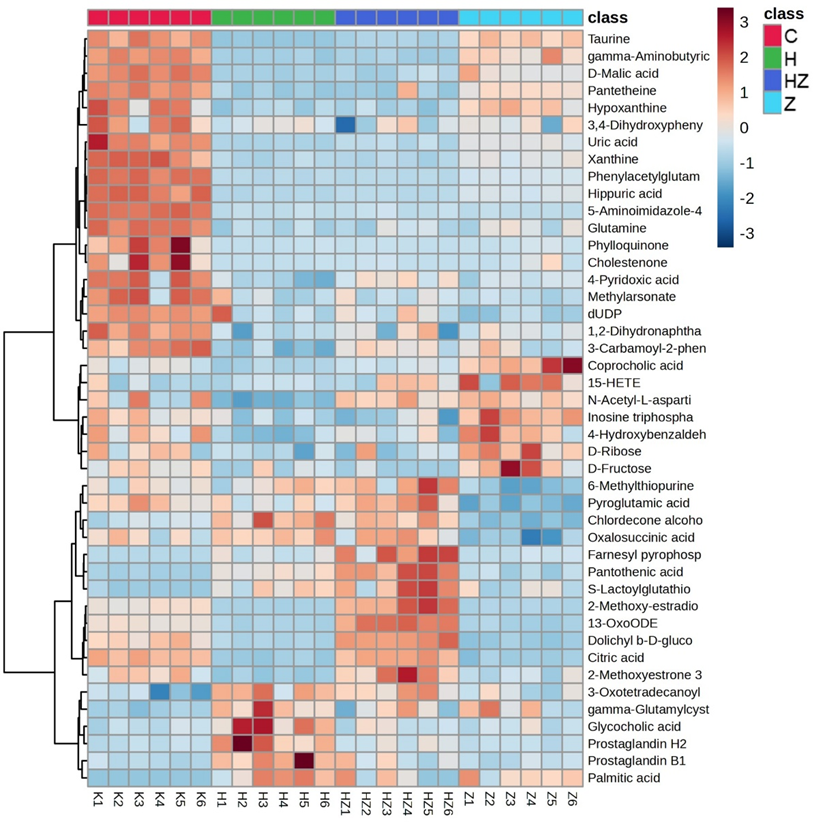


**Figure 7.** Heatmap analysis of metabolites significantly changing in HCQ, ZEA and combination groups in metabolomics analysis

**Figure 8.**

| VIP Scores  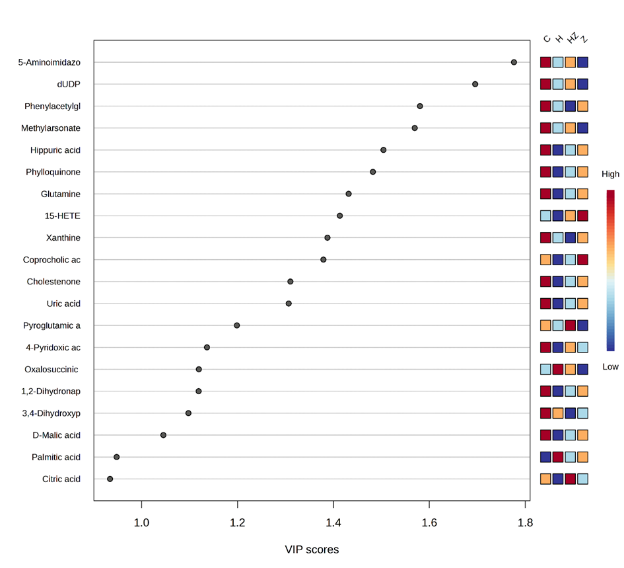 | HCQ  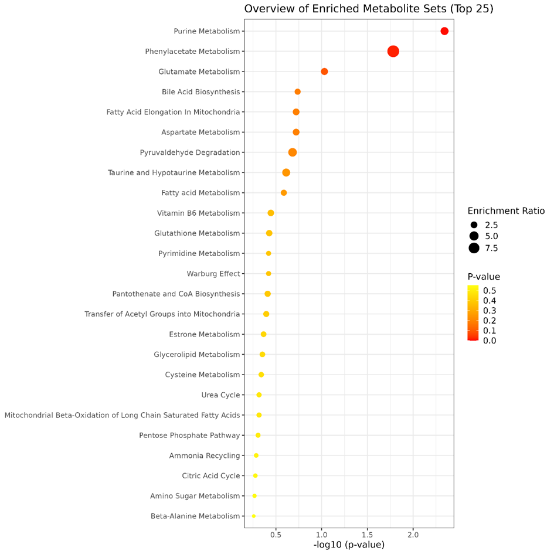 |
| --- | --- |
| ZEA  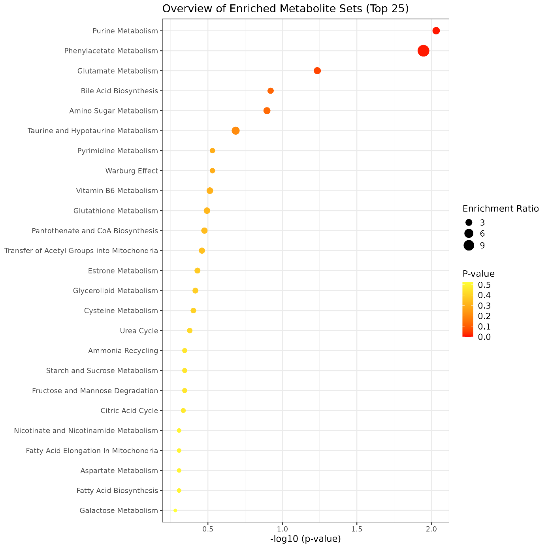 | HCQ+ZEA  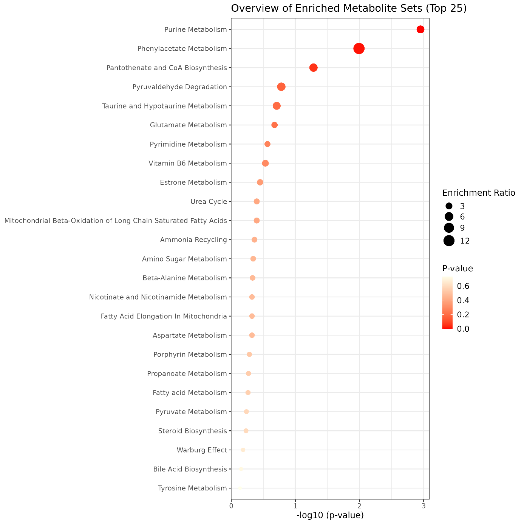 |

**Figure 8.** PLS-DA VIP scores and pathway enrichment analysis. Purine metabolism, phenylacetate metabolism, and pantothenate/coenzyme A biosynthesis were identified as the most significantly affected pathways (p < 0.05).

**Figure 9.**


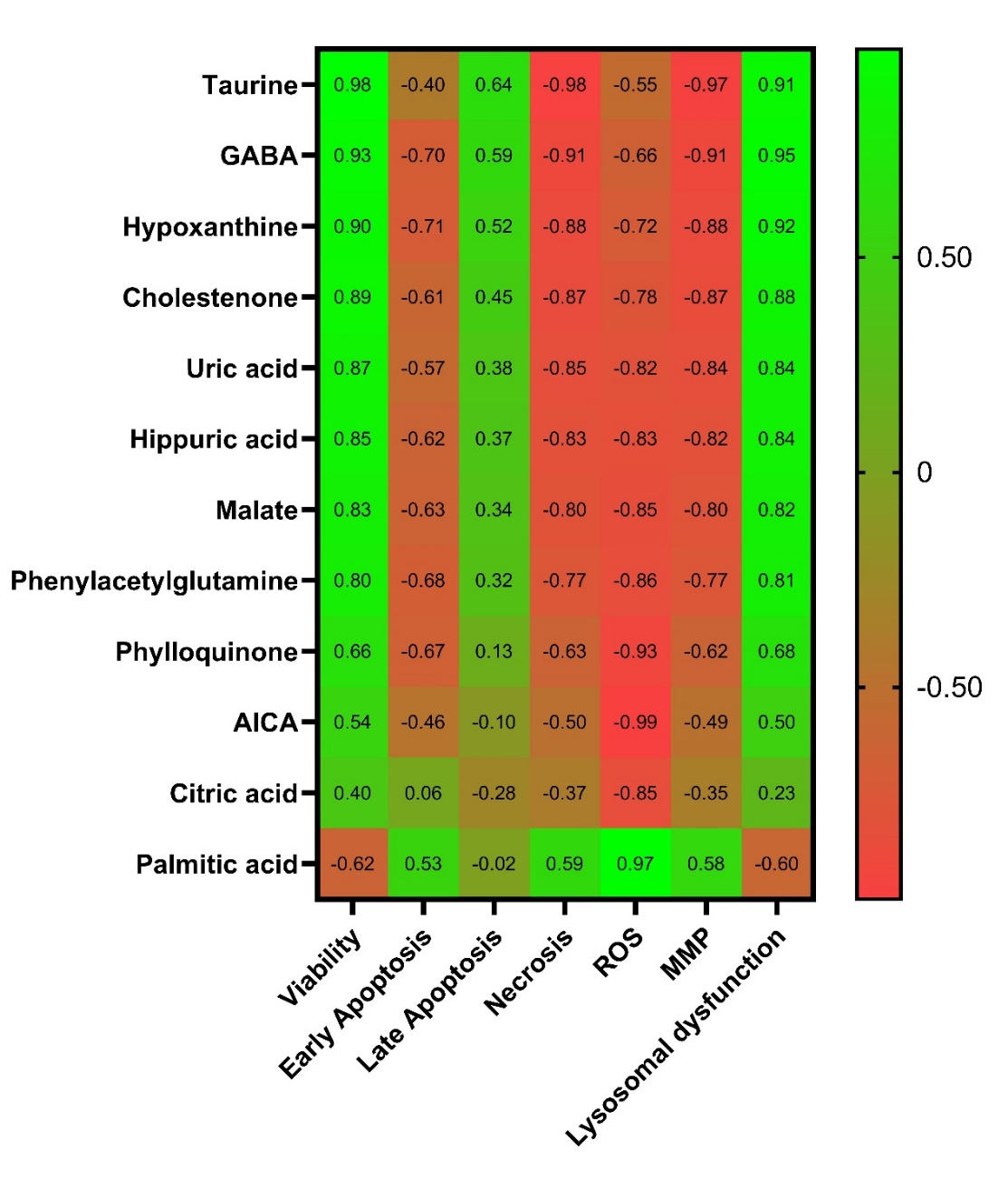


**Figure 9.** Pearson correlation analysis between functional parameters (viability, ROS, ΔΨm, AO ratio) and metabolite levels. Taurine, GABA, hypoxanthine, uric acid, and citrate were positively correlated with viability and negatively correlated with ROS and ΔΨm loss. Palmitic acid was positively correlated with ROS.

**Supporting Figure 1.**


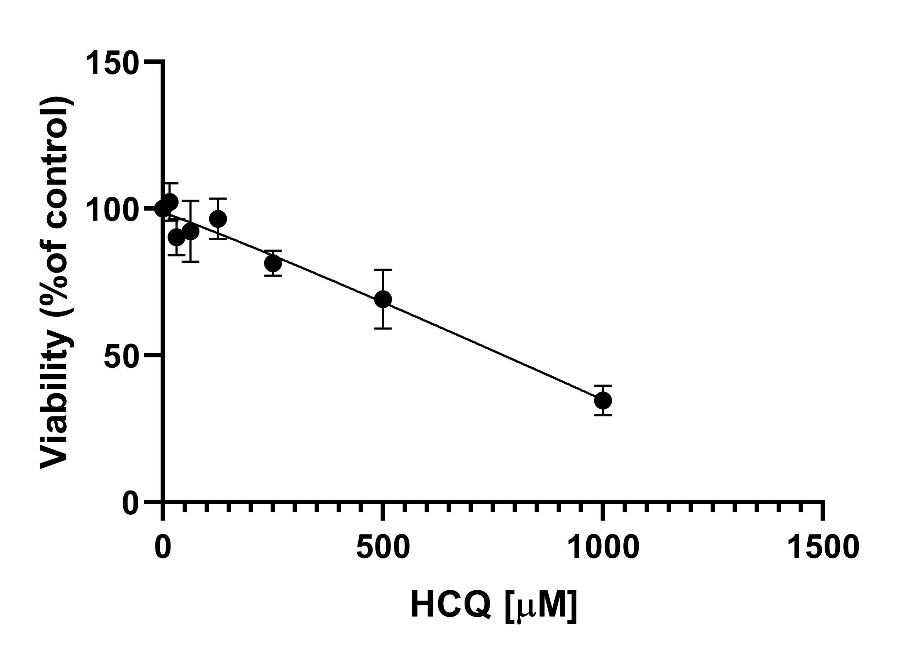


**Supporting Figure S1**. Dose-dependent effect of HCQ on ARPE-19 cell viability after 72 hours of treatment. Cells were exposed to various concentrations of HCQ (15.6–1000 µM), and viability was assessed by MTT assay. Data are presented as mean ± SE (n=3).
